# Different effect of CVVHDF and coupled plasma filtration and adsorption on IL-6 and procalcitonin in sepsis

**DOI:** 10.1186/cc9537

**Published:** 2011-03-11

**Authors:** F Turani, M Falco, R Barchetta, F Candidi, A Marinelli, C Di Corato

**Affiliations:** 1 European Aurelia Hospital, Rome, Italy

## Introduction

A decrease of IL-6 and procalcitonin (PCT) correlates with survival during sepsis [1]. Coupled plasma filtration and adsorption (CPFA) supports the renal function and removes proinflammatory mediators, but few clinical studies compare the effects of CPFA and CVVHDF, the standard of care in septic patients with renal failure [2]. The aim of this study is to evaluate whether CPFA and CVVHD have a different effect on IL-6 and PCT in septic patients.

## Methods

Seventy septic patients have been enrolled in this study. Fifty-five patients were submitted to CPFA. Every patient had four CPFA treatments (LINDA; Bellco-Mirandola, Italy) for 8 hours with Qb = 200 ml/minute, Q ultrafiltration = 30 ml/kg/hour and Q plasma = 20% of Qb. Fifteen septic patients submitted to CVVHDF were used as the control group. At T0 (basal), T1 (after 24 hours), T2 (after 76 hours), plasma IL-6 and plasma PCT was evaluated. ANOVA was used to compare changes during times study. *P *< 0.05 was considered statistically significant.

## Results

Tables 1 and 2 present the main results of this study. In the CPFA group at T2 IL-6 and PCT decreased to lower levels than T0, whereas in CVVHDF no significant change was observed. Hemodynamic data and adrenergic support improved more in the CPFA group than in the CVVHDF group.

**Table 1:** IL-6 and procalcitonin during CPFA

CPFA	T0	T1	T2
IL-6 (pg/ml)	393 ± 87	235 ± 56	113 ± 23*
Procalcitonin (ng/ml)	23 ± 9	16 ± 5	5 ± 2*

*P < 0.001 between T2 and T0.

**Table 2:** IL-6 and procalcitonin during CVVHDF

	T0	T1	T2
IL-6 (pg/ml)	262 ± 67	433 ± 96	144 ± 35
Procalcitonin (ng/ml)	18 ± 6	17 ± 7	14 ± 4

## Conclusions

CPFA seems more efficient then CVVHDF to remove either IL-6 or PCT and to improve hemodynamic status. Further studies are warranted to show whether these data may translate into a better clinical outcome.
